# First-Principles Study of χ_3_-Borophene as a Candidate for Gas Sensing and the Removal of Harmful Gases

**DOI:** 10.3390/nano13142117

**Published:** 2023-07-20

**Authors:** Jia-Xing Duan, Yu-Ping Tian, Chao-Bo Wang, Lian-Lian Zhang

**Affiliations:** College of Sciences, Northeastern University, Shenyang 110819, China

**Keywords:** borophene, adsorption, harmful gas, first-principles calculation

## Abstract

The potential application of borophene as a sensing material for gas-sensing devices is investigated in this work. We utilize density functional theory (DFT) to systematically study the adsorption mechanism and sensing performance of χ_3_-borophene to search for high-sensitivity sensors for minor pollutant gases. We compare the results to those for two Pmmn borophenes. The first-principles calculations are used to analyze the sensing performance of the three different borophenes (2 Pmmn borophene, 8 Pmmn borophene, and χ_3_-borophene) on five leading harmful gases (CO, NH_3_, SO_2_, H_2_S, and NO_2_). The adsorption configuration, adsorption energy, and electronic properties of χ_3_-borophene are investigated. Our results indicate that the mechanism of adsorption on χ_3_-borophene is chemisorption for NO_2_ and physisorption for SO_2_ and H_2_S. The mode of adsorption of CO and NH_3_ on χ_3_-borophene can be both physisorption and chemisorption, depending on the initially selected sites. Analyses of the charge transfer and density of states show that χ_3_-borophene is selective toward the adsorption of harmful gases and that N and O atoms form covalent bonds when chemisorbed on the surface of χ_3_-borophene. An interesting phenomenon is that when 8 Pmmn borophene adsorbs SO_2_, the gas molecules are dismembered and strongly adsorb on the surface of 8 Pmmn borophene, which provides a way of generating O_2_ while adsorbing harmful substances. Overall, the results of this work demonstrate the potential applications of borophene as a sensing material for harmful gas sensing or removal.

## 1. Introduction

The extraction of fossil fuels, such as oil, gas, and coal, has contributed considerably to meet the increasing global energy demand. However, these fuels generate large quantities of harmful gases [1]. Industrial exhaust gases can be categorized into particulates, gas, and radioactive pollutants. These combustion products have caused numerous global problems, such as the greenhouse effect, the hole in the ozone layer, acid rain, and widespread environmental pollution [2,3]. Hence, it is vital to remove these harmful gases from the environment. 

Two-dimensional (2D) materials are considered to be nanomaterials with a sheet-like morphology featuring a large lateral size ranging from hundreds of nanometers to tens of micrometers or, even more significantly, a thickness of a single or a few atomic layers [4,5]. Two-dimensional materials with unusual properties are some of the most promising candidates for numerous applications, including electronics, optoelectronics, catalysis, energy storage, solar cells, biomedicine, sensors, environments, etc. [6]. Due to the larger available surface volume ratio of 2D materials, the adsorption of gas molecules can arouse significant signals in the sensor’s materials, leading to higher sensing performance [7,8].

In 2004, the discovery of graphene ushered in a new era in the study of 2D materials [9]. The large surface area and high conductivity of graphene have resulted in extensive applications [10], such as electrode materials, molecular and other types of sensors, hydrogen storage materials, and data storage [11]. Graphene is the material used most extensively from nanoelectronics to aerospace applications [12,13]. Borophene is structurally analogous to graphene and was first theoretically predicted by Tang et al. in 2007 [14]. Mannix et al. subsequently prepared borophene in the form of thin sheets on an Ag(111) substrate [15]. Bonding between boron atoms is more complex than between carbon atoms [15]; 16 allotropic compounds based on B_12_ have been discovered that can be synthesized by various methods. Compared to carbon, boron has a lower molecular weight and is more substantial, flexible, lighter, and more prone to chemical reactions. Thus, boron has tremendous potential as a sensor material. Previous density functional theory (DFT) calculations have demonstrated that single-atomic-layer boron sheets consisting of triangular and hexagonal motifs are locally stable, where the most stable structure is known as an α sheet [14]. Subsequent calculations led to the prediction of two novel 2D boron phases with nonzero thickness and higher durability than the α sheet [16]. The first of these phases is 8 Pmmn borophene (referred to as a β sheet), which has an electronic band structure with a distinct Dirac cone. This phase has a curved structure with eight atoms in the unit cell and is considerably more complex than other 2D materials, such as graphene and phosphorene [10,17]. The second phase is 2 Pmmn borophene (referred to as a γ sheet). Free-standing relaxation of this structure eliminates the slight corrugation along the A direction and preserves buckling along the B direction [15]. Calculations of the phonon spectra of the β and γ phases have been found to consist of only accurate frequencies; that is, both structures are mechanically stable [18,19]. Liquid-phase exfoliation has been used to synthesize β_12_-and χ_3_-borophenes with graphene-like hexagonal vacancies that exist stably and freely [20,21,22]. Systematically, Zhang et al. investigated the 2D boron sheet using cluster expansion, consisting of hexagonal holes and triangular shapes on group 11 elements used for chemical vapor deposition (CVD) growth [23]. In recent years, three types of boron nanosheets, i.e., δ_6_-borophene, β_12_-borophene, and χ_3_-borophene, have been successfully synthesized in experiments by choosing growth substrates of the noble metals Ag or Au. Because χ_3_-borophene has been synthesized in experiments, we chose it as the main research candidate. The other point is that the sensing performance of χ_3_-borophene on harmful gas small molecules is currently unclear. Considering that the structure of χ_3_-borophene is not complex compared to other borophene structures, we predict that it can have unique transmission characteristics and sensing performance. In this work, we select the other two slightly complex borophenes (2 Pmmn and 8 Pmmn) for comparison.

A series of studies have been carried out on the adsorption of harmful gases. For example, Leenaerts et al. used first-principles calculations to study the adsorption of H_2_O, NH_3_, CO, and NO on graphene. All the gases were physisorbed on graphene, and only weak charge transfer occurred between the small gas molecules and graphene [24,25]. The adsorption of small gas molecules on other 2D materials has also been extensively studied, including phosphorene [26,27], and germanene [28]. Many research groups have reported the adsorption behavior of gases on borophene, including SO_2_ [29], AC [30], HCN [31]^,^ H_2_S [32], HCOH [33], AD [34], DMA/TMA [35], and N2-containing gases (such as NO, NO_2_, NH_3_) [36,37]. Valadbeigi et al. reviewed DFT studies on the adsorption of CO, N_2_, NO, and other molecules on the boron cluster B_36_ [38]. DFT predictions have shown that the boron cluster B_36_ is a promising adsorbent for CO and NO molecules. Liu et al. used a first-principles study to explore adsorption by borophene of gas molecules (CO, CO_2_, NH_3_, NO, NO_2_, and CH_4_) [39]. Thus, borophene has broad prospects for the adsorption of harmful gases. Nevertheless, the results of research studies to date are insufficient to understand the adsorption of toxic gases on borophene.

Given the current understanding of borophene adsorption summarized above, first-principles calculations are performed in this study to investigate the characteristics of χ_3_-borophene adsorption of harmful gases, and the results are compared to those of the two Pmmn borophenes. We investigate the adsorption of five toxic industrial gases (CO, NH_3_, SO_2_, H_2_S, and NO_2_) by the three borophenes (2 Pmmn borophene, 8 Pmmn borophene, and χ_3_-borophene). The borophenes’ geometric structure and binding energy are calculated to determine the stable forms of the borophenes during the gas adsorption process. The calculation results show that adsorption on χ_3_-borophene is more effective and stable than on the Pmmn borophenes. Therefore, we further study χ_3_-borophene and find that physisorption and chemisorption of CO and NH_3_ occur at different sites on χ_3_-borophene. The adsorption of SO_2_ and H_2_S on χ_3_-borophene is physical. Adsorption of NO_2_ on χ_3_-borophene is chemical, indicating that χ_3_-borophene has a good detection ability for NO_2_. Chemisorption deforms a χ_3_-borophene monolayer, enhancing the interaction between the adsorbed molecules and the adsorbent surface. The phonon spectra, charge transfer, total density of states (TDOS), and partial density of states (PDOS) are calculated to analyze the interaction between the adsorbed gas and original borophene. The results of this study provide theoretical guidance for the practical application of χ_3_-borophene as an adsorbent and sensor for pollutant gases.

## 2. Computational Methods

All calculations are performed based on the first-principles calculations using the projector-augmented wave method implemented in the Vienna ab initio Simulation Package (VASP) [40]. Within the implemented DFT framework, the generalized gradient approximation (GGA) is used to correct the exchange-correlation functional between electrons, and the Perdew–Burke–Ernzerhof (PBE) function is used to describe the exchange and correlation energy [41,42]. Van der Waals forces are included by applying a dispersion-corrected framework (DFT-D3) [43]. The electron wave function is spread out as a plane wave with an energy cutoff of 400 eV to ensure convergence. To ensure the credibility of the calculation results, we model the χ_3_-borophene prototype and the borophene after cell expansion, using periodic boundary conditions in three-dimensional space and the 20 Å thickness for the vacuum layer in the z direction to avoid the interaction between occasional images. The Brillouin zone (BZ) of the three borophenes is sampled using 1 × 2 × 1, 3 × 2 × 1, and 3 × 4 × 1 mesh points in the k-space base on the Monkhorst–Pack scheme [44]. Structural optimization is performed to relax the structure until the change in the energy and Hellmann–Feynman forces acting on the structure is less than 1.0 × 10^−8^ eV/atom and 0.02 eV/Å, respectively. The adsorption systems consist of a 2 × 4 × 1 χ_3_-borophene supercell, a 5 × 4 × 1 2 Pmmn borophene supercell, and a 2 × 2 × 1 8 Pmmn borophene supercell. To evaluate the stability of adsorption and bonding, we calculate the adsorption energy ($E_{ad}$) using the following equation:

(1) $$E_{ad}=E_{X+Borophene}-E_{Borophene}-E_{X}.$$

$E_{X+Borophene}$, $E_{Borophene}$, and $E_{X}$ are the total energy of borophene with the adsorbed gas molecule, the original borophene, and the isolated gas molecules, respectively. In addition, we calculate the charge transferred from borophene to a gas molecule using the Bader charge analysis code [45]. The transferred charge reflects the change in the electron density between a gas molecule and the borophene surface during the interaction, which is calculated using the following equation:

(2) $$∆\rho =\rho_{X+Borophene}-\rho_{Borophene}-\rho_{X},$$

where $\rho_{X+Borophene}$, $\rho_{Borophene}$, and $\rho_{X}$ represent the total charge density of borophene with the adsorbed gas molecule, the original borophene, and an isolated gas molecule, respectively.

## 3. Results and Discussion

### 3.1. Geometric and Electronic Structures of Pristine χ_3_-Borophene

**Figure 1:**
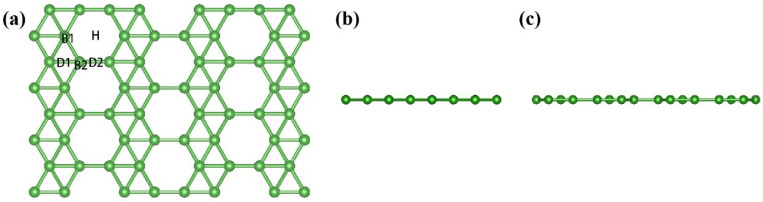
Top view of χ_3_-borophene along the (**a**) c-axis (**b**) a-axis, and (**c**) b-axis.

Figure 1 shows the top view of χ_3_-borophene along different directions. The χ_3_-borophene 2 × 4 × 1 supercell contains 64 atoms. The optimized lattice constants of the primitive cell were calculated and are shown in Table 1. The optimized B1-B1, B2-B2, B1-B2 bond lengths were 1.64 Å, 1.62 Å, and 1.71 Å, respectively, in agreement with the previously reported results [46]. The coordination numbers of B1 and B2 were 5 and 4, respectively. The optimized χ_3_-borophene was flat, without ripples along the A and B directions, and spliced by a triangular and hexagonal lattice. Several sites for adsorbing harmful gases were selected to determine the optimal adsorption configuration. These sites are shown in Figure 1a, where B, D, and H represent the top, bridge, and middle vacancy points, respectively. 

**Figure 2:**
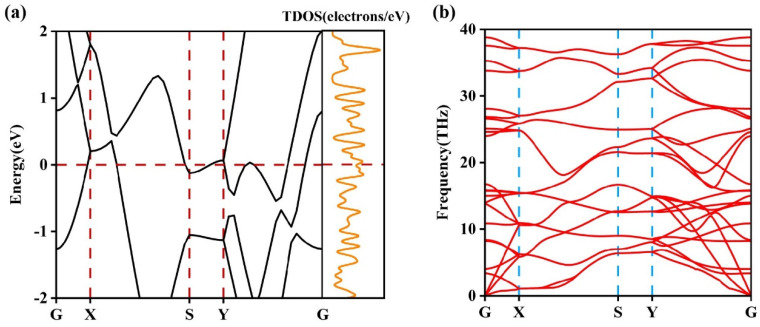
(**a**) Electronic band structure and TDOS of χ_3_-borophene. (**b**) Phonon spectrum of χ_3_-borophene. The Fermi energy is set to 0 eV.

Figure 2a shows the calculated band structure of χ_3_-borophene, where the high symmetry points follow a G-X-S-Y-G route in the reciprocal space of the BZ. From the electronic band structure and TDOS, one can find the typical metallic behavior and apparent anisotropy for χ_3_-borophene. At the Fermi level, χ_3_-borophene has a density of state (DOS) of 2.417 per eV. In the X-S and Y-G directions, multiple electron energy bands pass through the Fermi level, where the main contributions are from the p_y_ and p_z_ orbitals of the B atom. However, in the S-Y direction, the electron bands near the Fermi level are relatively flat, and the electronic states are more localized, indicating that the electronic properties vary with the directions. Figure 2b shows the phonon spectrum along several highly symmetric paths, where no imaginary frequency arises in the BZ. Hence, the χ_3_-borophene structure is stable. The optical phonon branch has a high eigenvalue of 38.89 THz, whereas the eigenvalue of graphene is 47.98 THz [48], showing that χ_3_-borophene is dynamically stable. The bond strength between boron atoms is comparable to that of the C-C bond.

### 3.2. Adsorption of Gases on Pristine χ_3_-Borophene

#### 3.2.1. Analysis of the Overall Trend of Gas Adsorption for Five Gases

The bond lengths of CO, NH_3_, NO_2_, SO_2_, and H_2_S are known to be 1.13 Å, 1.01 Å, 1.20 Å, 1.448 Å, and 1.543 Å, respectively. A monolayer of χ_3_-borophene was established onto which different harmful gases were adsorbed. The distance from the bottom borophene was set to 2 Å to control the variable. 

We selected five adsorption sites: B1, B2, D1, D2, and H. The five investigated gases are simple compounds composed of two elements. Therefore, each position selected two adsorption methods. The configuration in which the atom corresponding to the first element in the molecular gas formula of the gas was closer to χ_3_-borophene than the other atom was represented by −1. The configuration in which the atom corresponding to the second element in the molecular formula of the gas was closer to χ_3_-borophene than the other atom was represented by −2. Therefore, a total of ten adsorption configurations were investigated. Table 2 shows the adsorption energy, number of transfer electrons, and distance to the bottom borophene for the harmful gases adsorbed at different sites on χ_3_-borophene. An appropriate sensor requires both sensitivity and selectivity. When the structure of the adsorbed gas has sufficient charge transfer and appropriate adsorption energy, it proves that χ_3_-borophene can be used as an application sensor for detecting harmful gases.

Table 2 shows that the vertical adsorption of the CO molecules on the B (B1 and B2) and D (D1 and D2) sites shifted the χ_3_-borophene layer up. The B-B bond length changed from 1.71 Å to 1.79 Å, forming a C-B bond. These results mean an enormous interaction force exists between χ_3_-borophene and the C atom. When the O atom was closer to χ_3_-borophene, it always deflected the structure at this time. The distance between the CO molecule and the bottom borophene increased to approximately 3.3 Å, and the corresponding adsorption energy was approximately −0.1 eV. When the CO molecule was adsorbed on χ_3_-borophene with the C atom closer to the χ_3_-borophene structure than the O atom, the distance between the CO and the bottom borophene decreased to approximately 1.5 Å, resulting in the higher adsorption efficiency (−0.44~−0.685 eV). The adsorption of CO vertically on the H site did not lead to considerable rotation of the gas molecule. However, the distance between the gas molecules and the bottom borophene increased to approximately 3.1 Å, and the adsorption energy was relatively low. The highest adsorption energy (−0.685 eV) was obtained for adsorption on B2 with the C atom closer to χ_3_-borophene than the O atom, which was, therefore, the most stable configuration for the CO adsorption on the χ_3_-borophene layer.

At the B1, B2, D1, and D2 sites, when the N atom was closer to χ_3_-borophene (−0.538~−0.764 eV), the adsorption effect was remarkable, and the height was closer to χ_3_-borophene (about 1.64 Å). During chemisorption, the B atom moved upward, and the B–B bond increased in length (from 1.71 Å to 1.79 Å). However, when NH_3_ adsorbed on χ_3_-borophene at the H site, the height of the gas molecule above the χ_3_-borophene surface was slightly different, and the adsorption energy was approximately −0.134 eV. The adsorption on the D1 and D2 sites was more unstable than adsorption on the B1 and B2 sites, during which the gas molecules rotated slightly and shifted. The adsorption process of the NH_3_ was consistent with the adsorption process of the CO. The highest adsorption energy for NH_3_ (−0.764 eV) was obtained for the B2 site.

When χ_3_-borophene adsorbed the NO_2_, the adsorption on all sites was chemisorption. As the N atom in the initial NO_2_ configuration was closer to the χ_3_-borophene than the O atom, the final optimized structures for adsorption at the H, B2, D1, and D2 sites consisted of N-O bonds parallel to the D2, N-B bonds, and O-B bonds. As a result of the high adsorption energy (−2.067~−2.073 eV), the distance between the gas molecules and the bottom borophene decreased to approximately 1.55 Å. When the NO_2_ molecules adsorbed on the B1 site, with the N atom closer to the χ_3_-borophene than the O atoms, the optimized structure remained unchanged, but the adsorption was strong (−1.245 eV). Adsorption of the five selected sites with the O atoms closer to χ_3_-borophene than the N atom resulted in the formation of O-B bonds. Compared to the results for adsorption with the N atom closer to χ_3_-borophene than the O atoms, the NO_2_ adsorption shifted from B1 to D1, from B2 to D2, and from H to the left. This result showed that the NO_2_ adsorption on χ_3_-borophene was stronger when the N atoms were closer to χ_3_-borophene than the O atoms. High energy is required for NO_2_ to adsorb on the χ_3_-borophene surface relative to that required for adsorption on graphene [39] and blue-black phosphorene [49].

The adsorption of SO_2_ on χ_3_-borophene occurred via physisorption at all sites. For the direct adsorption at the H site, the bond angle of the small gas molecule did not change, but the gas molecules were far from the bottom of χ_3_-borophene (approximately 3.0 Å), and the adsorption energy was minimal. The adsorption was strongest when the S atom was closer to the χ_3_-borophene than the O atoms, indicating that the interaction force between the χ_3_-borophene and the S atom was higher than that between the χ_3_-borophene and the O atom. The angle between the S and O atoms deflected when the gas molecules adsorbed on the bridge sites (D1 and D2). On the other hand, when the SO_2_ adsorbed on the B site (B1 or B2), the angle deflection and movement of the gas molecule along the a- and b-axis occurred, and the gas molecule moved above the bridge site, suggesting that the adsorption on the B site (B1, B2) was unstable. 

Similarly, the adsorption of H_2_S on χ_3_-borophene occurred via physisorption at all sites. The optimization of the adsorption at the aforementioned ten sites resulted in almost no movement of the gas molecule along the ab plane and an indiscernible deflection of the H_2_S bond angle. When adsorption occurred with the H atom closer to the χ_3_-borophene than the S atom, the distance between the H atom and the borophene bottom ranged between 2.40 Å and 2.71 Å, and the adsorption energy ranged between −0.074 eV and −0.081 eV. By comparison, when adsorption occurred with the S atom closer to the borophene surface than the H atom, the distance between the H atom and the bottom borophene was shorter (3.24~3.43 Å), and the adsorption energy was higher (−0.032~−0.040 eV).

In summary, CO and NH_3_ adsorbed onto χ_3_-borophene by both physisorption and chemisorption. When a CO molecule adsorbed at a B site (B1 or B2) or a bridge site D (D1 or D2), the adsorption was stronger when the C atom was closer to the bottom borophene than the O atom (C > O). Similarly, the adsorption of NH_3_ was stronger when the N atom was closer to the bottom borophene than the H atom (N > H). Chemisorption occurred when the C and N atoms were closer to the bottom borophene than the O and H atoms, respectively, whereas physisorption occurred when the O and H atoms were closer to the bottom borophene than the C and N atoms, respectively. Finally, the adsorption was stronger at the B site than at the bridge site (B > D).

In addition, both the SO_2_ and H_2_S adsorbed on the χ_3_-borophene by physisorption. The adsorption of SO_2_ was stronger when the S atom was closer to the bottom borophene than the O atoms (S > O). Similarly, the adsorption of H_2_S was stronger when the H atom was closer to the bottom borophene than the S atom (H > S). Finally, the adsorption was stronger at the bridge site than at the top site (D > B). Last, NO_2_ adsorbed on the χ_3_-borophene by chemisorption. In the optimized final structure, the N-O bonds oriented parallel to D2 to form N–B and O–B bonds. The adsorption was stronger at the bridge site than at the top site (D > B) and when the gas molecule was closer to the surface than farther away (2 > 1). Finally, when the absolute value of the adsorption energy was smaller, the adsorption was relatively weaker. The optimization of the gas molecule configuration increased the distance between the optimized gas molecule and the bottom χ_3_-borophene, indicating a minor interaction between the gas molecule and χ_3_-borophene. 

#### 3.2.2. Electronic Structure of a System of a Gas Molecule Adsorbed on χ_3_-Borophene for Five Different Gases

**Figure 3:**
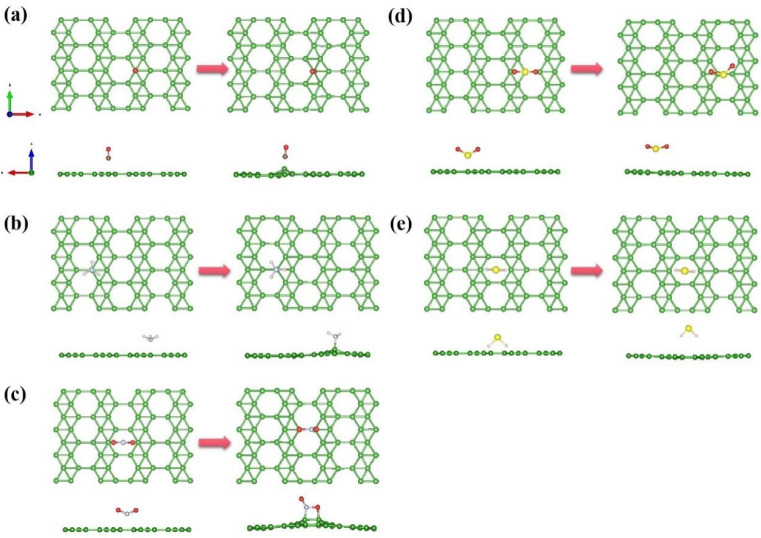
(**a**) Pristine and optimized structures for adsorbing five gases by χ_3_-borophene; (**a**) CO; (**b**) NH_3_; (**c**) NO_2_; (**d**) SO_2_; (**e**) H_2_S.

Based on the data for adsorption on χ_3_-borophene given in Table 2 for the pairs of gas configurations, we selected a representative set of structures for each gas (highlighted in bold in the table). Figure 3 shows the adsorption sites that optimized the system. Chemisorption of gas occurs via the formation of chemical bonds between the gas and χ_3_-borophene, which can cause the χ_3_-borophene to deform [50]. The deformation of χ_3_-borophene can enhance the interaction between the gas molecule and the χ_3_-borophene surface. By contrast, physisorption does not induce changes in the χ_3_-borophene structure. 

Next, we present the contributions of DFT and DFT-D3 to the adsorbed gases and plot the adsorption energy data in Table 3. The system we studied involved weak interaction, while the traditional DFT method has some shortcomings in describing the dispersion interaction. The DFT-D3 considers the geometric information of the structure to calculate the dispersion correction energy, which can reasonably predict the energy of the Van der Waals system. After using such two frameworks to calculate the harmful gases adsorbed by the χ_3_-borophene species, we found that the energy obtained by DFT-D3 was in better agreement with the charge transfer results. Therefore, we considered the DFT-D3 in the adsorption system.

**Figure 4:**
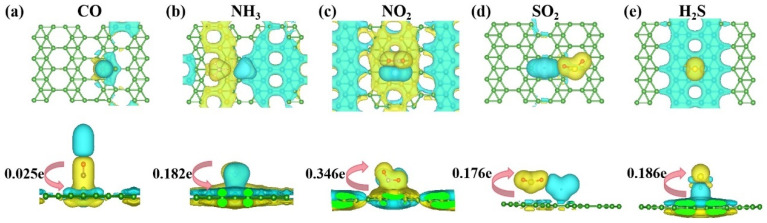
Differential charge density diagram of χ_3_-borophene.

Next, we analyzed the charge transfer, electronic band structure, and TDOS of the optimal adsorption sites for the aforementioned five gases. First, we performed a Bader charge analysis to study the stability of the χ_3_-borophene system further. Figure 4 shows a differential charge density plot of χ_3_-borophene, where the yellow and blue regions correspond to the charge accumulation and depletion, respectively. The yellow region around the small gas molecules adsorbed on χ_3_-borophene indicated that charge accumulated near the gas molecules. Figure 3 and Table 2 show that the charge transfer of only 0.025e occurred from χ_3_-borophene to CO. This result suggests that the main interaction between χ_3_-borophene and CO is the Van der Waals interaction, which proves that physisorption was the adsorption mechanism. A qualitative analysis of the differential charge density map of CO molecules above B1 indicates that the charge depletion is likely to occur for adsorption at this site. By comparison, NH_3_ and H_2_S are more likely to adsorb above H, whereas NO_2_ and SO_2_ are more likely to adsorb above D2. Different gases are likely to lose electrons at different positions because of the difference in the gas molecular structures and interactions between each atom in the gas and the B atom. For example, as N–B and O–B bonds form easily, the B atoms of χ_3_-borophene bonded with the N and O atoms of NO_2_ at the D2 site. 

**Figure 5:**
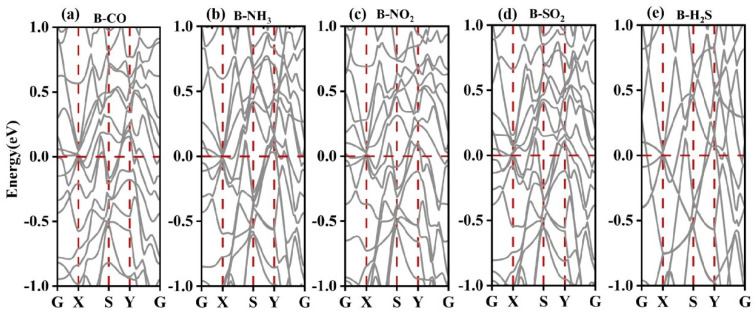
Electronic band structures after the adsorption of five gases by χ_3_-borophene.

Figure 5 shows an electronic band structure plot. For χ_3_-borophene with an adsorbed gas molecule, all the bands passed through the Fermi level, as in the case of χ_3_-borophene without adsorbed gas. Therefore, there was no gap in the band structure of χ_3_-borophene with an adsorbed gas molecule (for all five gases), indicating a metallic behavior. For NO_2_, NH_3_, or CO adsorption, the gas molecules interacted with the occupied and unoccupied electronic states of χ_3_-borophene far from the Fermi level. Thus, the molecules of these three gases had almost no effect on the electronic properties of χ_3_-borophene near the Fermi level.

**Figure 6:**
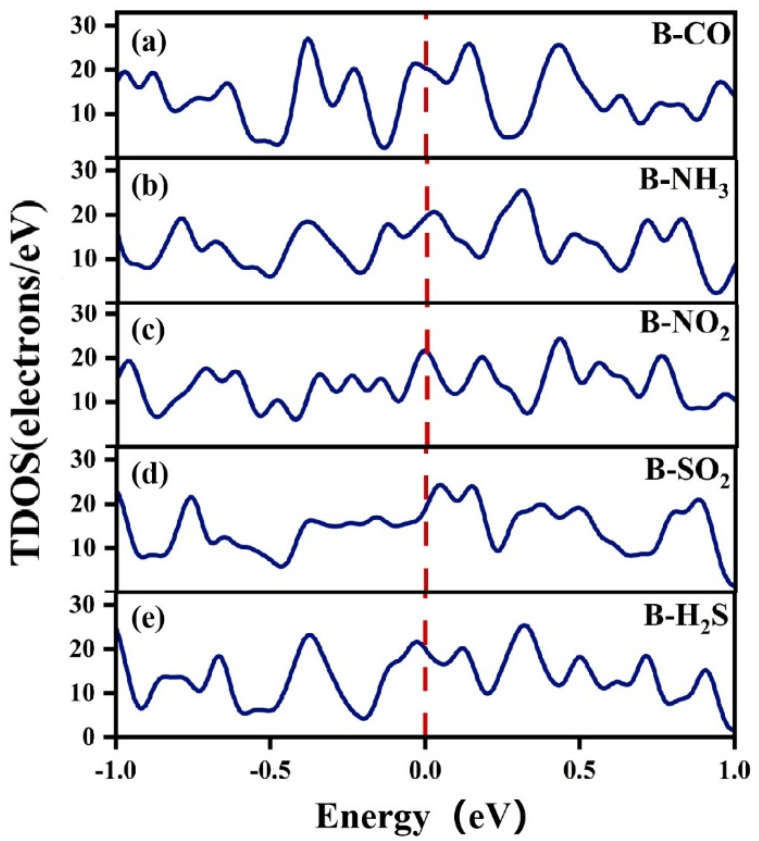
TDOS after the adsorption of five gases by χ_3_-borophene.

Figure 6 shows the TDOS diagrams of χ_3_-borophene after the adsorption of a gas molecule for the five gases. The TDOS crossed the Fermi energy level for all five adsorbed gases, proving that adsorption did not change the metallic properties of χ_3_-borophene. The DOS between the two peaks near the Fermi level was not zero for all five gases. This pseudo energy gap directly reflected the covalence of bonding in the χ_3_-borophene–adsorbed gas system. The strongest covalent bonding was observed for the adsorption of NO_2_. 

**Figure 7:**
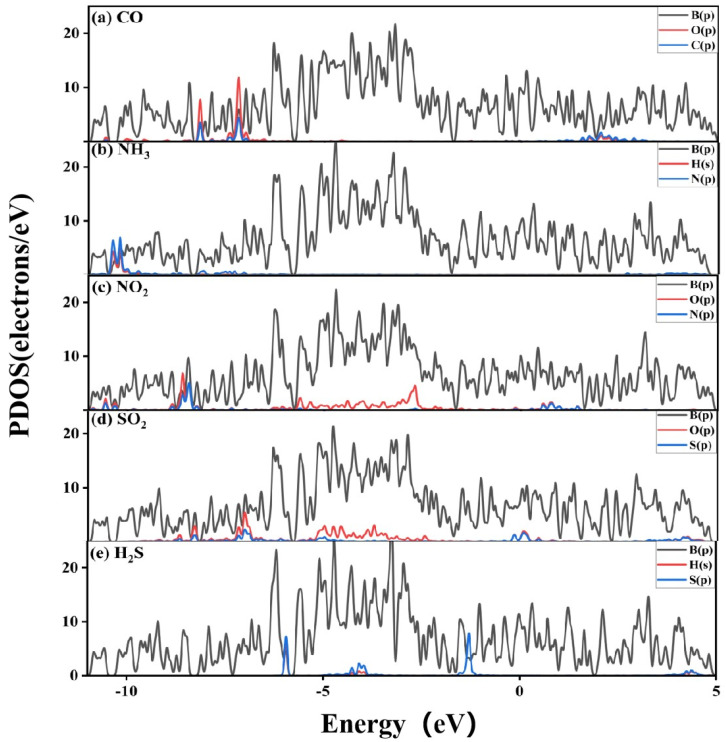
PDOS diagram after the adsorption of five gases by χ_3_-borophene (**a**) CO; (**b**) NH_3_; (**c**) NO_2_; (**d**) SO_2_; (**e**) H_2_S.

Figure 7 shows the PDOS diagram to determine the contributions of individual atoms to the electronic properties of the χ_3_-borophene–adsorbed gas system. The p-orbital of the B atom mainly contributed to the five TDOS near the Fermi level. The PDOS diagram of CO-χ_3_-borophene revealed that the DOS peaks of the p-orbitals of the B atom (B-p) overlapped with those of the C-p and O-p atoms in the −6~−8 eV range of the valence region. It can therefore be understood that χ_3_-borophene-p interacted with the O and C atoms. 

We then focused on the PDOS of the gas molecules near the Fermi level. It was found that the B, C, and O atoms all contributed to the TDOS near the Fermi level. In the PDOS diagram of NH_3_-χ_3_-borophene, the DOS peak of the B-p orbit overlapped with the N-p and H-s orbit in the −10~−12 eV range of the other valence region. This indicates that the p-orbital of χ_3_-borophene interacted weakly with the N and H atoms. In the PDOS diagram of NO_2_-χ_3_-borophene, the orbitals overlapped in the −8 to 3 eV range. The N-p and O-p orbits transitioned from the conduction band to the valence band near the Fermi level, which confirmed that charge transfer from the χ_3_-borophene surface to the NO_2_ molecules caused enhanced metallic properties of the NH_3_-χ_3_-borophene system. In the PDOS diagram of SO_2_-χ_3_-borophene, the broadening and shift of the peak in the range of −8~2 eV was caused by the electron transfer from χ_3_-borophene to the SO_2_ molecule, leading to the overlap of the B-p, S-p, and O-p orbits. Gas adsorption increased the TDOS at the Fermi energy level and enhanced the metallic properties of χ_3_-borophene for all configurations of the adsorbed gas molecule. In the PDOS diagram of H_2_S-χ_3_-borophene, there were several peaks in the range of −6 to −1 eV over which the S-p orbital interacted with the B-p orbital that all occurred in the valence region. In addition, SO_2_ contributed to the Fermi energy state, which may affect the conductivity of SO_2_-χ_3_-borophene.

The Fermi energy level was increased, and the metallic properties of χ_3_-borophene were enhanced by the adsorption of all five gases. The electronic structure analysis shows that χ_3_-borophene has broad prospects as a gas sensor.

### 3.3. 2-Pmmn Borophene and 8 Pmmn Borophene

**Figure 8:**
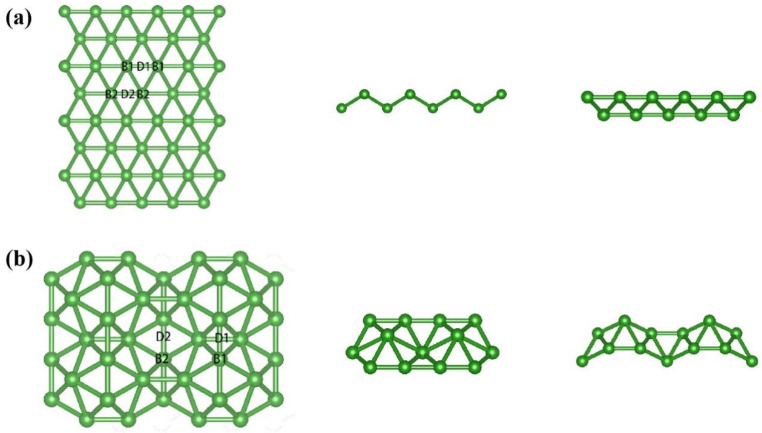
(**a**,**b**) The top view of 2 Pmmn borophene and 8 Pmmn borophene along the c-axis direction, along the a-axis path, and the b-axis approach is shown in sequence.

We also studied the adsorption of harmful gases by the two other types of borophene, 2 Pmmn and 8 Pmmn. The adsorption by χ_3_-borophene of the toxic gases was good relative to the other two borophenes. The lattice constant of Pmmn borophene is well-matched with the (110) surface of certain metals or metal oxides. Thus, Pmmn borophene can be synthesized by depositing boron atoms on specific metal substrates. It is known that graphene has been experimentally prepared by this method [10]. Figure 8 shows the structures of 2 Pmmn and 8 Pmmn borophene. The 2 Pmmn borophene is the most studied type of borophene and has no ripples along the a-axis direction and a W-shaped ripple structure along the b-axis with a considerable buckling height. Calculation of the energy band and density of states shows that 2 Pmmn borophene exhibits strong anisotropic metallic properties, which can induce facile electron transfer and electrical conduction at room temperature. However, the absence of ripples along the a-axis direction limits the conductivity of 2 Pmmn borophene.

Moreover, 8 Pmmn borophene is a zero-gap semiconductor. The density of states at the Fermi level is zero. In the band structure, there is a Dirac cone, and the valence band and conduction band meet at the junction point (0, 0.3, 0) at the Fermi level. We investigated five adsorption sites on 2 Pmmn and 8 Pmmn borophene: B1, B2, D1, and D2. The same nomenclature was used for χ_3_-borophene; that is, the configuration in which the atom corresponding to the first (second) element in the molecular formula of the gas was closer to borophene than the other atom in the gas molecule was represented by −1 (−2). Eight adsorption mechanisms were considered for each type of borophene. The adsorption energy and charge transfer number of 2 Pmmn and 8 Pmmn borophene for the absorption of harmful gases at different sites are shown in Table 4 and Table 5, respectively.

**Figure 9:**
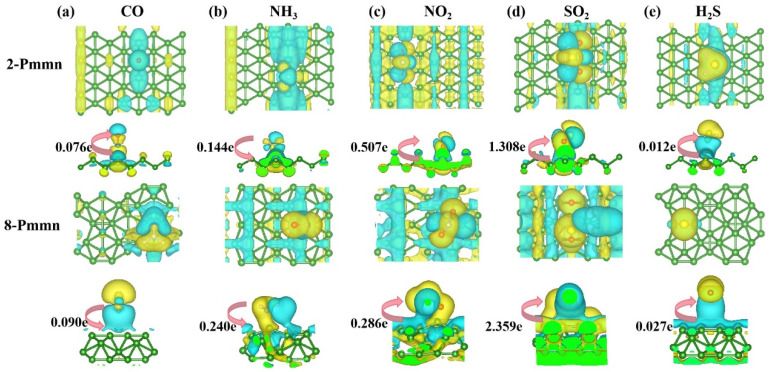
Differential charge density diagrams of (up) 2 Pmmn borophene and (down) 8 Pmmn borophene. (**a**) Results of the CO; (**b**) NH_3_; (**c**) NO_2_; (**d**) SO_2_; (**e**) H_2_S.

The results in the two tables presented above clearly demonstrate that these two borophenes can adsorb harmful gases, and the adsorption site determined the magnitude of the adsorption energy. Also, the physisorption results from relatively weak interactions between a gas molecule and the absorbent surface. Physisorption is nonspecific and involves relatively weak van der Waals forces and low adsorption energies. In addition, physically adsorbed molecules can diffuse along the surface of an adsorbent and are usually not bound to specific locations on the surface. Because the gas molecules are only weakly bound to the adsorbent surface, physisorption can be rapidly reversed. The chemical bond can be created by the sharing of electrons between the adsorbate and the adsorbent and can be regarded as the formation of a surface compound. Chemisorption is difficult to reverse, because of the strong adhesion between the adsorbate and adsorbent [51]. However, as chemisorbed gas molecules cannot be easily desorbed into the gas phase, boron cannot be reused after the gas has been adsorbed onto borophene. Raw materials are thus wasted. Figure 9 shows the results of the differential charge density analysis of the two Pmmn borophenes for the adsorption of (from left to right) CO, NH_3_, NO_2_, SO_2_, and H_2_S. 

In Figure 9, the sizable blue area below the gas molecule after adsorption by 2 Pmmn borophene indicates that borophene lost electrons. The small gas molecule was surrounded by yellow regions, indicating that the gas received electrons. The quantity of charge transferred can be used to preliminarily determine the type of adsorption involved. After a gas molecule was adsorbed on the 8 Pmmn borophene, a “#”-shaped blue area appeared high above the borophene surface. By contrast, a “#”-shaped yellow area appeared below the B atom at the bottom of borophene. An intriguing result is that the absolute value of the adsorption energy of the 8 Pmmn borophene for SO_2_ was considerably higher than those of the other two borophenes, indicating chemisorption. The optimized result after adsorption was S–O bond breakage, meaning that 8 Pmmn borophene may be able to dismember toxic gas molecules. It is speculated that this behavior results from the migration of half of the electrons in 8 Pmmn borophene from the interior to the bridge B atom, transforming 8 Pmmn borophene into a covalent single-element 2D material with ionic properties. Compared to planar borophene, 8 Pmmn borophene is more stable and therefore less prone to deformation. This property may enable 8 Pmmn borophene to dismember and thereby adsorb SO_2_ more effectively than planar borophene. The fracture of the chemical bond can occur via a free radical reaction, which can be realized by ionization or electron transfer. The cleavage of SO_2_ molecular bonds could occur via charge transfer, resulting in a more robust and stable O–B bond for 8 Pmmn borophene than for planar borophene. 

After a comparative analysis of the three types of borophene, it was found that the adsorption capacity decreased in the order of 2 Pmmn borophene > χ_3_-borophene > 8 Pmmn borophene. This result suggests that borophene with metallic properties has better adsorption performance than borophene with semiconductor properties. The site most prone to electron loss was the H for the χ_3_-borophene and the B1 for the 2 Pmmn and 8 Pmmn borophene. The adsorption mechanism determines the adsorption energy for small gas molecules. Vertical adsorption was most efficient for CO because the C atoms were located closer to the borophene surface than the O atoms. The most efficient NH_3_ adsorption occurred when the N atoms were closer to the borophene surface than the H atoms. The most efficient NO_2_ adsorption occurred when the O atoms were closer to the borophene surface than the N atoms. The most efficient SO_2_ adsorption occurred when the O atoms were closer to the borophene surface than the S atoms for 2 Pmmn and 8 Pmmn borophene) but when the S atoms were closer to the borophene surface than the O atoms for χ_3_-borophene. The adsorption of H_2_S was most efficient when the H atoms were closer to the borophene surface than the S atoms for the χ_3_- and 8-Pmmn borophene but when the S atoms were closer to the borophene surface than the H atoms for 2 Pmmn borophene.

**Figure 10:**
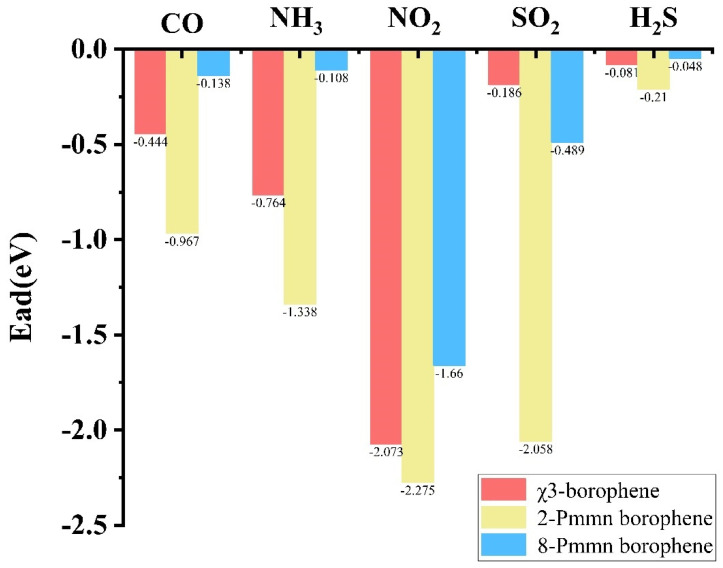
Comparison diagram of the three systems adsorbing harmful gases.

As shown in Figure 10, if the adsorption energy is too high, it will cause small molecules of gas and borophene to tightly adsorb in the form of chemical adsorption, leading to the waste of raw materials and turning borophene into a disposable sensor device. If the adsorption energy is too low, it will lead to very unstable adsorption, and once there is a slight change in the external environment, it will lead to the desorption of gas on the adsorption. In addition, especially when 8 Pmmn borophene adsorbs gas, the difference in the adsorption energy at each site is too large, so it can be inferred that its adsorption of harmful gases is very unstable. Thus, compared to the other two Pmmn borophenes, without the excessive waste of raw materials, χ_3_-borophene has a better ability to adsorb harmful gases, and the gas structure after the adsorption by χ_3_-borophene is relatively stable.

## 4. Summary

In summary, we performed a first-principles calculation to investigate the adsorption potentials and effects of harmful gas molecules (CO, NH_3_, NO_2_, SO_2_, and H_2_S) on three types of borophenes and then determined the most efficient adsorption site and mechanism. Our used lattice constant of χ_3_-borophene was almost consistent with the previous experimental report, which ensured the correctness of our structure and provided a good basis for the following experiments. Compared to the two Pmmn borophenes, χ_3_-borophene was found to have a better ability to adsorb harmful gases and can be used without excessive waste of raw materials. The adsorption capacity of χ_3_-borophene was different for the five gases and was strongest for NO_2_ because a covalent bond formed between the NO_2_ and χ_3_-borophene. The high energy and large charge transfer of χ_3_-borophene for gas adsorption makes χ_3_-borophene a candidate material for gas sensor applications. However, chemisorption results in the waste of raw materials, because χ_3_-borophene cannot be reused and becomes a disposable sensor device. The adsorption mechanism for H_2_S and SO_2_ on χ_3_-borophene was pure physisorption, which requires low adsorption energy but a high transfer charge. This result indicates that χ_3_-borophene–adsorbed gas structures are relatively stable after adsorption. Therefore, χ_3_-borophene is a good adsorbent. As CO and NH_3_ can be both physisorbed and chemisorbed on χ_3_-borophene, χ_3_-borophene has high selectivity and is, therefore, a good choice for adsorbing these gases. In addition, it has been found that the adsorption of SO_2_ by 8 Pmmn borophene occurs by the decomposition of the gas molecules followed by the strong adsorption of the atoms on the surface of 8 Pmmn borophene, which could be exploited to generate O_2_ during the adsorption of harmful substances. All the results obtained in this work demonstrate that χ_3_-borophene has broad prospects as a gas sensor for adsorbing toxic gases.

## Figures and Tables

**Table 1:** The lattice constants for χ_3_-borophene.

	a/Å	b/Å
This work	8.48	2.94
Ref. [47]	8.42	2.90

**Table 2:** The adsorption energy, transfer electron number, height, and structural changes of the adsorption of harmful gases at different sites on χ_3_-borophene.

	Position	B1-1	B1-2	B2-1	B2-2	H-1	H-2	D1-1	D1-2	D2-1	D2-2
CO	$E_{ad}(eV)$	**−0.444**	−0.087	−0.685	−0.104	−0.091	−0.083	−0.498	−0.103	−0.532	−0.121
	Δq	**0.025e**	0.043e	0.034e	−0.055e	−0.066e	0.053e	0.058e	0.008e	0.052e	0.050e
	$d_{bottom}(Å)$	**1.51**	3.48	1.50	3.31	3.15	3.05	1.55	3.50	1.53	3.42
NH_3_	$E_{ad}(eV)$	−0.538	−0.124	**−0.764**	−0.138	−0.134	−0.135	−0.758	−0.118	−0.148	−0.129
	Δq	0.191e	0.148e	**0.182e**	−0.147e	−0.011e	0.167e	0.197e	0.142e	0.023e	0.158e
	$d_{bottom}(Å)$	1.65	2.97	**1.64**	2.73	2.86	2.82	1.64	2.94	2.72	2.82
NO_2_	$E_{ad}(eV)$	−1.245	−1.559	−2.071	−2.221	**−2.073**	−1.436	−2.072	−1.555	−2.067	−2.175
	Δq	0.450e	0.438e	0.348e	−0.450e	**−0.346e**	0.481e	0.352e	0.439e	0.356e	0.447e
	$d_{bottom}(Å)$	1.61	1.48	1.55	1.54	**1.54**	1.47	1.56	1.48	1.55	1.52
SO_2_	$E_{ad}(eV)$	−0.183	−0.058	**−0.186**	−0.145	−0.112	−0.037	−0.185	−0.193	−0.178	−0.176
	Δq	0.168e	0.315e	**0.176e**	−0.339e	−0.108e	0.117e	0.176e	0.187e	0.164e	0.158e
	$d_{bottom}(Å)$	2.95	2.27	**2.96**	2.34	2.88	3.00	2.98	2.98	2.91	2.95
H_2_S	$E_{ad}(eV)$	−0.080	−0.039	−0.081	−0.038	**−0.081**	−0.040	−0.080	−0.032	−0.074	−0.035
	Δq	0.180e	0.004e	0.184e	−0.001e	**−0.186e**	0.006e	0.176e	0.001e	0.195e	0.003e
	$d_{bottom}(Å)$	2.49	3.43	2.40	3.34	**2.58**	3.24	2.70	3.43	2.71	3.34

**Table 3:** Calculation of the system for adsorbing harmful gases on χ_3_-borophene using the DFT-D3 and DFT methods.

$E_{ad}(eV)$	CO	NH_3_	NO_2_	SO_2_	H_2_S
DFT-D3	−0.444	−0.764	−2.073	−0.186	−0.081
DFT	−0.323	−0.577	−1.933	−0.003	0.788

**Table 4:** The adsorption energy, transfer electron number, height, and structural changes of the adsorption of harmful gases at different sites on 2 Pmmn borophene.

	Position	B1-1	B1-2	B2-1	B2-2	D1-1	D1-2	D2-1	D2-2
CO	$E_{ad}(eV)$	−0.762	−0.113	−0.163	−0.120	−0.113	−0.114	**−0.967**	−0.121
	Δq	0.142e	−0.046e	0.053e	−0.062e	0.034e	0.045e	**0.076e**	−0.061e
NH_3_	$E_{ad}(eV)$	−1.332	**−1.333**	−0.230	−0.230	−1.334	−0.224	−0.230	−0.229
	Δq	0.152e	**0.144e**	0.159e	−0.152e	0.151e	0.130e	0.153e	−0.155e
NO_2_	$E_{ad}(eV)$	−1.712	**−2.275**	−1.712	−2.156	−1.711	−2.178	−1.713	−2.158
	Δq	0.349e	**−0.507e**	0.409e	−0.356e	0.367e	0.438e	0.424e	−0.357e
SO_2_	$E_{ad}(eV)$	−0.132	**−2.058**	−0.100	−0.111	−0.121	−1.095	−0.168	−0.423
	Δq	0.106e	**−1.308e**	0.097e	−0.067e	0.109e	1.250e	0.481e	−1.672e
H_2_S	$E_{ad}(eV)$	−0.105	−0.226	−0.231	−0.231	−0.106	**−0.210**	−0.243	−0.245
	Δq	0.093e	0.049e	0.047e	−0.026e	0.102e	**0.012e**	0.027e	0.034e

**Table 5:** The adsorption energy, transfer electron number, height, and structural changes of the adsorption of harmful gases at different sites on 8 Pmmn borophene.

	Position	B1-1	B1-2	B2-1	B2-2	D1-1	D1-2	D2-1	D2-2
CO	$E_{ad}(eV)$	**−0.540**	−0.070	−0.138	−0.108	−0.051	−0.054	−0.110	−0.109
	Δq	**0.090e**	−0.030e	0.054e	−0.066e	0.011e	0.030e	0.063e	−0.063e
NH_3_	$E_{ad}(eV)$	**−0.707**	−0.108	0.193	−0.160	−0.086	−0.084	−0.168	−0.164
	Δq	**0.240e**	−0.099e	0.205e	−0.211e	0.810e	0.871e	0.197e	−0.715e
NO_2_	$E_{ad}(eV)$	−1.253	−1.251	−0.706	−0;813	−1.255	**−1.660**	−0.601	−0.503
	Δq	0.333e	−0.346e	0.475e	−0.463e	0.334e	**0.286e**	0.968e	−0.798e
SO_2_	$E_{ad}(eV)$	−0.071	−0.094	−0.467	−0.110	0.868	−0.090	−0.489	**−0.489**
	Δq	0.079e	−0.093e	1.282e	−0.095e	0.763e	0.090e	2.383e	**−2.359e**
H_2_S	$E_{ad}(eV)$	−0.049	−0.023	−0.127	−0.119	**−0.048**	−0.016	−0.119	−0.074
	Δq	0.021	0.006	0.025e	−0.082e	**0.027e**	0.001e	0.026e	0.001e

## Data Availability

The data presented in this study are available on request from the corresponding author.
